# Special Issue “Brain Injury: New Insights into Mechanisms and Future Promising Treatments”

**DOI:** 10.3390/biomedicines13051190

**Published:** 2025-05-13

**Authors:** Kristina Pilipović, Petra Dolenec

**Affiliations:** Department of Basic and Clinical Pharmacology and Toxicology, Faculty of Medicine, University of Rijeka, 51000 Rijeka, Croatia; kristina.pilipovic@medri.uniri.hr

This Special Issue of *Biomedicines* presents 12 published, peer-reviewed articles on the theme of “Brain Injury: New Insights into Mechanisms and Future Promising Treatments”, including 10 original research papers and 2 reviews covering the topics related to the pathophysiology of acquired central nervous system (CNS) injuries, as well as biomarker discovery, diagnosis, and potential pharmacological and non-pharmacological approaches in the treatment of these disorders.

Each year, upwards of 85 million people sustain acquired brain injuries, the most common of which are traumatic injuries and stroke. The treatment of injured CNS tissue, due to its structural and functional complexity and slow regeneration rate, presents a particular challenge for medical practitioners. Most of the therapeutic approaches in brain injury management that have been studied in the preclinical and clinical settings have been focused on trying to reduce the consequences of initial injury and enhance the function of the remaining brain and spinal cord tissue. However, there is still the unmet need to regenerate or replace damaged or necrotic tissue. One promising approach for the repair of traumatically injured brains involves using nanotechnology and tissue engineering approaches, techniques that focus on bridging structural gaps and allowing the reconnection of severed neuronal processes.

In this Special Issue, we aimed to cover recent research from preclinical and clinical studies and present relevant and up-to-date reviews, thus highlighting the current knowledge and opportunities for future tailored research in this area.

Traumatic brain injury (TBI) refers to damage to the brain caused by a sudden mechanical force to the skull. It is a significant public health concern globally, affecting millions each year and often resulting in long-term disability [1]. The immediate damage caused by TBI, as well as the secondary pathophysiological changes within the CNS, can have life-long consequences [2]. The severity of TBI is categorized as mild, moderate, or severe, with mild TBI, commonly referred to as a concussion, being the most prevalent form. However, even mild TBI can result in chronic health issues, particularly when it occurs repeatedly, as is common in contact sports or among military personnel [3,4,5,6].

While mild TBIs are generally temporary, moderate and severe injuries often result in more significant neurological impairments, requiring intensive neurorehabilitation. Notably, both single and repetitive TBIs are associated with an increased risk of neurodegenerative diseases, such as Alzheimer’s disease, Parkinson’s disease, and chronic traumatic encephalopathy [4,7,8,9,10]. A common pathological marker among these conditions is the aggregation of the TAR DNA-binding protein 43 (TDP-43) [11]. Research conducted by Janković et al. [12] explored the effects of both moderate and repetitive mild TBIs on TDP-43 expression in the cervical spinal cord of mice. The findings indicate that repetitive mild TBI leads to significant increases in TDP-43 aggregation and microglial activation, suggesting a detrimental long-term impact on the CNS.

In addition to the immediate damage caused by mechanical forces, TBI often triggers a range of secondary injury processes, including an elevation in intracranial pressure (ICP), cerebral edema, and hypoxia. Hernandez et al. [13] explored the role of cathepsin B (Cath B) in the progression of TBI in a rat model, particularly under conditions of elevated ICP. Previous research has shown that elevated ICP following TBI exacerbates neuronal membrane disruption [14]. Cath B is a lysosomal protease implicated in different TBI pathologies, e.g., cell damage and death processes [15,16], and it seems to have a role in the exacerbation of behavioral morbidities following TBI [17,18]. While direct effects of Cath B on membrane disruption were not conclusively established in the study by Hernandez et al. [13], their results suggest a potential involvement of Cath B in TBI-induced sensory hypersensitivity. This finding warrants further research into Cath B’s role in TBI progression and provides compelling evidence that inhibiting Cath B may serve as a viable neuroprotective strategy in TBI management.

TBI is often accompanied by hydrocephalus, a condition characterized by the impaired flow of cerebrospinal fluid (CSF) and ventricular enlargement [19]. It often leads to significant neurological impairments, including gait disturbance, cognitive deficits, and urinary incontinence [19,20,21,22]. However, in many patients with hydrocephalus, bladder dysfunction more commonly appears as storage-related symptoms, including frequent urination, urgency, and detrusor overactivity, rather than true incontinence [23]. The periaqueductal gray and the locus coeruleus are the key brain regions involved in the regulation of the micturition reflex, and changes in these regions are associated with bladder dysfunction in hydrocephalus [23]. Loucano et al. [24] investigated hydrocephalus in a rat model. Their findings suggest that urinary dysfunction in hydrocephalus may stem from disrupted descending noradrenergic modulation, pointing to significant neurological consequences due to the damaged brainstem circuits.

Post-traumatic stress disorder (PTSD) is a psychological condition that can arise following exposure to traumatic events. While PTSD is typically associated with emotional and psychological trauma, there is growing evidence suggesting that it can also be linked to neurological injury, including TBI [24,25,26]. PTSD symptoms, such as intrusive memories, flashbacks, and hyperarousal, are common among individuals who have sustained TBI, particularly mild TBI [27]. Gillam et al. [28] reviewed the relationship between TBI and PTSD. Their work highlights the bidirectional relationship between these conditions, emphasizing the role of neuroinflammation and altered stress response mechanisms. The review suggests that future research should focus on integrative therapeutic approaches that address both neurological and psychological aspects of TBI-related PTSD.

The COVID-19 pandemic has introduced new challenges in understanding the neurological implications of viral infections [29,30,31]. Mavroudis et al. [31] explored post-COVID-19 neurological syndrome (PCNS) and its similarities to post-concussion syndrome, highlighting overlapping symptoms such as fatigue, cognitive difficulties, and mood and sleep disturbances. Their review identifies shared mechanisms such as neuroinflammation, autonomic dysfunction, and prolonged cognitive deficits. These findings suggest that the lessons learned from TBI research could inform therapeutic approaches for PCNS, particularly in individuals experiencing long-term cognitive impairment post-COVID-19.

Disorders of consciousness (DoCs), such as coma, minimally conscious state, or unresponsive wakefulness syndrome, are common consequences of TBI. Recovery from DoC is typically assessed through behaviors like responding to stimuli or following commands [32]. Gangemi et al. [33] explored the potential of transcranial stimulation in modulating neuroplasticity following TBI. Using rodent models, they demonstrate significant improvements in cognitive recovery, supporting the therapeutic promise of non-invasive brain stimulation in TBI rehabilitation. Their findings showed that a combination of robotic verticalization training and transcranial direct current stimulation enhances motor function and hemodynamic stability in individuals with DoC.

Foot drop syndrome (FDS) is a common condition in people with severe acquired brain injury (ABI); it is characterized by significant weakening and atrophy of the dorsiflexor muscles of the foot [34]. Piccione et al. [34] proposed a simplified electrophysiological screening for identifying the causes of FDS to help clinicians to recognize the significant clinical predictors of poor outcomes in severe ABI at admission to an intensive rehabilitation unit. Their electrophysiological screening method allows for the early identification of neuropathies associated with ABI, improving rehabilitative strategies for these patients. Their study demonstrates that precise diagnostic tools can facilitate early interventions, ultimately enhancing functional recovery and mobility in affected individuals.

The severity of TBI is often assessed using the Glasgow Coma Scale (GCS), which measures the level of consciousness following injury [35]. However, the predictive value of the GCS in terms of long-term outcomes remains limited [36,37]. Friedrich et al. [38] investigated the pathophysiology of TBI in a clinical cohort, analyzing inflammatory markers and their correlation with cognitive and motor deficits. Their findings suggest that targeting specific inflammatory pathways could offer new therapeutic avenues for TBI treatment. Specifically, they report that the C-reactive protein/albumin ratio serves as a potential biomarker for predicting in-hospital mortality in TBI patients, offering a valuable prognostic tool for clinical management. Furthermore, recent studies have shown that metabolites can be a crucial biomarker for TBI [39]. Detecting the changes in these metabolites may be significant for evaluating post-TBI neurological recovery [40]. Research has linked TBI severity to specific biomolecules, such as medium-chain fatty acids and certain phospholipids, but the exact role of these metabolites remains unclear [39,41,42]. Duan et al. [40] investigated the causal effect of serum metabolites and CSF metabolites on TBI susceptibility through Mendelian randomization. A metabolic pathway analysis found ten related pathways that played a role in the pathogenesis of TBI, which may help us to better comprehend the underlying mechanisms of TBI. These metabolites may serve as useful circulating biomarkers in clinical screening and prevention, and could also be candidate molecules for the exploration of mechanisms and drug targets.

Ischemic stroke, a condition resulting from the blockage of blood flow to the brain, leads to neuronal injury due to the lack of oxygen and glucose [43]. While research on neuroprotective strategies for ischemic stroke has advanced, there are still significant gaps in understanding the best therapeutic interventions [43]. Antonova et al. [44] explored the potential neuroprotective effects of krypton in ischemic brain injury. Noble gases like xenon, argon, and krypton have gained attention for their potential medical applications due to their ability to exert biological effects without being metabolized [45]. These gases have shown promise in activating the Nrf2 transcription factor, which plays a key role in managing oxidative stress and enhancing cellular resistance to damage [44]. In Antonova et al.’s research [44], krypton demonstrated neuroprotective effects in hypoxic conditions, improving survival rates in animal models exposed to low oxygen levels.

In addition to ischemic mechanisms, genetic factors also contribute to the severity and risk of stroke. Pavlov et al. [46] investigate hypoxic–ischemic encephalopathy (HIE), a severe brain injury caused by oxygen deprivation during birth, emphasizing genetic predispositions that influence disease progression. Their study identifies key genetic variants associated with HIE severity, paving the way for precision medicine approaches in neonatal brain injury. Their findings underscore the importance of early genetic screening to identify at-risk populations for targeted therapeutic strategies.

Aneurysmal subarachnoid hemorrhage leads to high mortality rates due to both the initial bleeding and delayed cerebral ischemia (DCI) [47,48,49]. Traditional treatments targeting vasospasms, such as nimodipine, have shown limited success in improving outcomes [50]. Hofmann et al. [50] examined cerebral ischemia and its neurobiological underpinnings. Their clinical study evaluates metabolic changes post-stroke, providing insights into potential metabolic targets for therapeutic intervention. They report that variations in microvascular perfusion contribute to DCI, emphasizing the need for multimodal treatment approaches such as induced hypertension and intra-arterial nimodipine to optimize patient outcomes.

The articles featured in this Special Issue collectively advance our understanding of the mechanisms of brain injury and identify promising therapeutic strategies. Clinical studies provide valuable insights into patient outcomes and potential prognostic markers, while preclinical investigations lay the foundation for novel treatment approaches. The review studies further contextualize emerging research trends, bridging gaps between clinical observations and mechanistic insights. Future research should continue integrating these findings to optimize translational applications and improve patient care. The diversity of approaches presented in this collection underscores the complexity of brain injury and the necessity of multidisciplinary collaboration in addressing this pressing medical challenge.
